# Molecular Docking and Pharmacological In Silico Evaluation of Camptothecin and Related Ligands as Promising HER2-Targeted Therapies for Breast Cancer

**DOI:** 10.3390/cimb47030193

**Published:** 2025-03-15

**Authors:** Elmer Joel Millan-Casarrubias, Yunia Verónica García-Tejeda, Claudia Haydée González-De la Rosa, Lucero Ruiz-Mazón, Yazmín Mariela Hernández-Rodríguez, Oscar Eduardo Cigarroa-Mayorga

**Affiliations:** 1 Department of Advanced Technologies, UPIITA-Instituto Politécnico Nacional, Av. IPN 2580, Mexico City 07340, Mexico; emillan@cinvestav.mx (E.J.M.-C.); ygarciat@ipn.mx (Y.V.G.-T.); yazmin.hernandez@cinvestav.mx (Y.M.H.-R.); 2 Departamento de Ciencias Naturales, Unidad Cuajimalpa, Universidad Autonóma Metropolitana, Mexico City 05348, Mexico; 3 Departamento de Biología Celular, Centro de Investigación y de Estudios Avanzados del Instituto Politécnico Nacional (CINVESTAV-IPN), Mexico City 07360, Mexico; lucero.ruiz@cinvestav.mx

**Keywords:** camptothecin, molecular docking, EGFR, HER2, breast cancer, pharmacokinetics

## Abstract

Breast cancer is one of the leading causes of cancer-related mortality in women worldwide, highlighting the importance of effective therapies. This study evaluates the interaction between camptothecin, a potent anticancer agent, and two key receptors implicated in breast cancer progression: HER2 (human epidermal growth factor receptor 2) and EGFR (epidermal growth factor receptor), using molecular docking. The results reveal a stronger binding affinity between camptothecin and HER2 than EGFR, in contrast to neratinib, which demonstrated affinity exclusively for HER2. Camptothecin exhibits significant hydrophobic and pi-alkyl interactions with HER2, whereas its interactions with EGFR are primarily mediated by hydrogen bonds. Molecular dynamics (MD) simulations of the camptothecin-HER2 complex indicate stable binding, with minimal fluctuations observed over 100 nanoseconds, confirming the stability of the ligand–receptor interaction. Pharmacokinetic evaluations, based on Lipinski’s rule of five, demonstrate that camptothecin adheres to essential drug-likeness parameters, suggesting favorable bioavailability. Furthermore, the analysis comparing the pharmacological properties of camptothecin with other well-known anticancer compounds, such as neratinib, shows that camptothecin exhibited superior compliance with drug-likeness rules. Despite its low solubility, the binding stability and pharmacokinetic profile suggest its potential as an effective therapeutic agent for breast cancer, particularly when combined with drug delivery systems that enhance solubility. This work underscores the importance of receptor-specific ligand interactions in drug design and highlights the need for further studies into camptothecin’s clinical applications, especially in HER2-positive breast cancer treatment.

## 1. Introduction

Breast cancer is a type of cancer with high incidence and mortality rates worldwide [1]. According to the World Health Organization (WHO), approximately 685,000 deaths due to breast cancer were reported in 2020 [2]. Different types of breast cancer have been identified, with the most common being invasive ductal carcinoma and HER2-positive and triple-negative breast cancer [3,4,5]. Among these, triple-negative breast cancer is the most complex and aggressive, with fewer treatment options available [6]. Various therapies have been developed for treating this type of cancer, tailored to the cancer subtype, disease stage, patient characteristics, and other conditions. The main treatment modalities include surgery, radiotherapy, chemotherapy, hormonal therapy, targeted therapy, and immunotherapy [5,6,7]. Despite these advances, the advantages and disadvantages of these treatments, combined with the high incidence and mortality rates, necessitate the development of more effective treatments with fewer side effects.

Treatment for HER2-positive breast cancer, while showing promise, has specific limitations. Resistance to therapy, such as the prolonged use of trastuzumab, can reduce drug efficacy over time. Other challenges include side effects, tumor recurrence, and tumor heterogeneity, as tumors of the same type may exhibit significant biological variation. Additionally, the high cost and limited accessibility of treatments pose significant barriers [8,9,10,11]. In this context, phytocompounds have gained increasing attention in recent years. These natural-origin compounds tend to have fewer or negligible adverse effects compared to synthetic drugs [12]. Phytocompounds have a wide range of applications, owing to their antioxidant, anti-inflammatory, anticancer, antimicrobial, cardio-protective, and metabolic regulation effects [13,14,15,16,17]. However, a significant limitation of phytocompounds is the challenge of their extraction and purification, which has prompted the development of various synthesis methods.

Among the numerous phytochemicals studied, camptothecin (CPT), a naturally occurring alkaloid derived from Camptotheca acuminata, has emerged as a powerful chemotherapeutic agent [18]. Camptothecin and its derivatives are well known for their ability to inhibit topoisomerase I, an enzyme crucial for DNA replication, transcription, and repair. By targeting topoisomerase I, camptothecin induces DNA damage in rapidly dividing cells, ultimately leading to apoptosis. Although camptothecin has demonstrated substantial efficacy in treating several types of cancer, including lung, ovarian, and colorectal cancer, its potential as a breast cancer therapeutic treatment remains underexplored [18,19]. In breast cancer studies, camptothecin has primarily been evaluated in combination with HER2-directed antibodies and not as a standalone agent, mainly due to its low solubility in aqueous environments. The main challenges associated with camptothecin use include its poor water solubility, undesirable side effects such as diarrhea and myelosuppression, and the potential for tumor resistance arising from prolonged administration [20,21,22].

Advances in computational approaches, such as molecular docking and molecular dynamics simulations, have revolutionized drug discovery and design, enabling the in silico prediction of molecular interactions between potential therapeutic agents and biological targets. These techniques facilitate the screening of vast compound libraries, identifying those most likely to bind specific protein targets involved in disease pathways. In cancer research, molecular docking can predict the affinity of anticancer compounds for critical receptors overexpressed in tumors, such as the epidermal growth factor receptor (EGFR) and human epidermal growth factor receptor 2 (HER2). Both receptors play pivotal roles in the pathogenesis of breast cancer. HER2-positive breast cancer represents an aggressive subtype characterized by poor prognosis and resistance to standard therapies. Identifying compounds that effectively target HER2 could provide new insights into therapeutic interventions for breast cancer. Several in vitro and in vivo studies have confirmed the anticancer potential of camptothecin derivatives. However, their effectiveness against specific breast cancer targets remains incompletely understood. In this context, in silico techniques can clarify the molecular mechanisms by which camptothecin interacts with breast cancer-associated receptors, including EGFR and HER2 [18,19].

The aim of this study was to analyze the anticancer potential of camptothecin against HER2 and EGFRs expressed in breast cancer, as a possible alternative treatment. Molecular docking and molecular dynamics simulations were employed to evaluate the interactions between camptothecin and two receptors overexpressed in breast cancer: EGFR and HER2 [23,24]. Camptothecin’s ability to inhibit these receptors could offer a dual-targeting approach, providing a therapeutic advantage in combating breast cancer [25]. Furthermore, the pharmacological properties of camptothecin were evaluated using Lipinski’s rule of five [26], which predicts a compound’s drug-likeness based on its molecular weight, lipophilicity (logP), hydrogen bond donors, and hydrogen bond acceptors. Lipinski’s rule is significant in several key areas, including predicting drug solubility and permeability, reducing costs and development time, enhancing the success rate of clinical trials, and providing a foundation for drug design [27,28]. These rules are commonly applied in the early stages of drug development to predict oral bioavailability and assess a compound’s suitability for clinical use [29,30]. Compliance with Lipinski’s rules is a critical step in identifying viable drug candidates and can guide chemical modifications or the development of drug delivery systems to enhance therapeutic efficacy [31].

Despite these promising findings, camptothecin’s poor solubility remains a significant barrier to clinical application. Novel strategies, such as nanomedicine-based formulations or the development of camptothecin prodrugs, have been proposed to address these limitations. Examples include solid lipid nanoparticles, nanoemulsions, liposomes, and polymeric nanoparticles [32,33,34]. Nanotechnology offers a promising solution by improving drug delivery, enhancing solubility, and reducing systemic toxicity. Several studies have demonstrated the efficacy of nanoparticle-based delivery systems for camptothecin, providing a potential pathway to overcome its pharmaco-kinetic challenges [35].

This study provides a comprehensive in silico evaluation of the potential of camptothecin as an anticancer agent for breast cancer. The results suggest that camptothecin is a promising candidate for targeting HER2-positive breast cancer. However, further studies are needed to address its solubility issues and optimize its delivery. These findings underscore the importance of computational techniques in modern drug discovery and highlight camptothecin’s potential for future therapeutic development.

## 2. Materials and Methods

### 2.1. Molecular Docking Procedure

All ligands were initially sketched and rendered using the BIOVIA Draw 2022 [36] software, which was employed to generate the 2D molecular structures. Once the 2D structures were generated, the next step involved generating the 3D geometries of the ligands.

Avogadro software [37] (https://sourceforge.net/projects/avogadro) (accessed on 11 January 2024) was used to convert the 2D structure into coordinates, followed by minimization using Gaussian 09W software [38]. This was accomplished using semi-empirical optimization through the PM3 method [39], implemented in Gauss View 5.0 software. This process is critical for ensuring that the ligands adopt realistic and energetically favorable 3D conformations, which are crucial for accurate molecular docking studies.

For the molecular docking, the protein structures of the two receptors, HER2 and EGFR, were obtained from the Protein Data Bank (PDB) (https://www.rcsb.org/) (accessed on 7 March 2024) [40]. Specifically, the HER2 receptor was represented by PDB code: 3PP0 [41], while the EGFR was assigned PDB code: 1M17 [42]. These PDB entries provided high-resolution 3D structures of the target receptors, serving as the basis for further computational studies. The structures of the two receptors were prepared by removing unnecessary ligands and water molecules, as well as adding hydrogen and the required charges. To this end, the Discovery Studio Visualizer 2021 [43] software and the Autodock Tools suite (version 1.5.7) (https://autodocksuite.scripps.edu) (accessed on 20 April 2024) [44] were used. To complete and model the missing amino acid residues, the CHARMM-GUI web server (https://www.charmm-gui.org/) (accessed on 10 June 2024) was used, following the protocol outlined in the PDB reader and manipulator section [45,46].

The docking simulations were conducted using the Autodock Tools suite (version 1.5.7), a widely used software for molecular modeling. A blind docking approach was employed, which means that the entire surface of the protein was targeted without preselecting any specific binding site. This method ensures that potential unknown or allosteric binding sites could be explored during docking, providing a comprehensive understanding of ligand–receptor interactions.

The docking simulations were performed using a Lamarckian genetic algorithm [47], with an initial population size of 100 individuals and a maximum number of energy evaluations set to 10,000,000. The ligand was treated as flexible, while the receptors were regarded as rigid. The grid box dimensions for the HER2 receptor were 126 × 126 × 126 Å, with a spacing of 0.502 Å, and the box was centered on the receptor. For the EGFR, the grid box dimensions were 100 × 72 × 58 Å, with a spacing of 1 Å, and the box was centered on the receptor. The genetic algorithm simulates evolutionary processes to identify the most stable ligand–receptor complex based on Gibbs free energy. The conformations with the lowest Gibbs free energy were selected as the most favorable binding poses and subjected to further analysis.

Visualization of the docking results, including the ligand–receptor interactions, was carried out using AutoDockTools 1.5.4 and the Discovery Studio Visualizer. These visualization tools were crucial for analyzing and presenting the interaction patterns, such as hydrogen bonds and hydrophobic interactions, between the ligands and their respective receptor targets. This comprehensive workflow, from structure optimization to visualization, allowed for a thorough investigation of the molecular interactions involved in the study.

### 2.2. Molecular Dynamic

The stability of the complexes derived from molecular docking was assessed using molecular dynamics techniques to calculate the RMSD (root mean square deviation) and RMSF (root mean square fluctuation) parameters. The complexes with the most favorable energy were selected, and using the BIOVIA Discovery Studio Visualizer program, the coordinates for both the ligand and the receptor were saved separately. The simulation was conducted using GROMACS software 2022.1 [48]. The Avogadro program was used to convert the coordinates to Sybyl mol2 format for building the ligand topology, followed by making some corrections in GROMACS. The CGenFF web platform (https://app.cgenff.com/homepage) (accessed on 18 July 2024) was subsequently used to create the topology for the ligand in Sybyl mol2 format. For the receptor topology, GROMACS was employed with the pdb2gmx command along with a CHARMM36 all-atom force field [49]. The complex was created by combining the coordinates of the ligand and receptor into a single file. The system was then solvated in a cubic box using the TIP3P and SPC216 models [50], ensuring a distance of 1.0 nm between the complex and the box. The system was neutralized by adding chlorine or sodium ions based on the total charge. The system underwent minimization and equilibration for 100 ps under constant NVT and NPT conditions, maintaining a temperature of 300 K and a pressure of 1.0 bar. Finally, the complex underwent a manufacturing process lasting 100 ns, during which the RMSD and RMSF of the protein backbone were measured. The procured results were then evaluated against the results obtained from the MDS of the unbound HER-2 or EGFR. The approximate number of frames per simulation was 5000.

### 2.3. Prediction of Pharmacological Properties

Various online tools were used to determine specific pharmacokinetic parameters. The Molinspiration server (https://www.molinspiration.com/cgi/properties) (accessed on 15 October 2024) was employed to assess the parameters of the Lipinski rules, including the number of acceptor and donor protons, Log P, and molecular weight. The SwissADME tool from the Swiss Institute of Bioinformatics web server (http://www.swissadme.ch/) (accessed on 11 September 2024) was used to predict pharmacological properties [51,52]. Two-dimensional ligands in the Sybyl mol2 data format were employed for uploading files to various platforms.

## 3. Results

### 3.1. Molecular Docking

#### 3.1.1. Coupling Energies (ΔG) and Dissociation Constants of Ligands Against the HER2 and EGFR Receptors

Table 1 presents the docking results obtained for the interaction between various ligands and the HER2 receptor, with Neratinib serving as a positive control due to its known affinity for the receptor. The ligands chosen for this study were selected because they are naturally occurring compounds found in certain plants and are, in some cases, already used to treat other conditions. This may result in a lower toxicity margin compared to synthetic substances, which require extensive studies to assess their safety for patient administration. Given the vast number of phytocompounds available, we also opted to include compounds with diverse structures to investigate any structure–activity relationships in relation to the evaluated receptors. The docking energy values, expressed as negative free energy, provide an indication of the strength of the interaction between each ligand and the receptor [53]. The more negative docking energy value, the stronger the binding affinity. The importance of this is that the free energy is directly related to the dissociation constant (Kd), where a more negative free energy corresponds to a lower dissociation constant. The inverse relationship between these two parameters means that the lower the Kd, the stronger the ligand’s affinity for the receptor.

In this context, the dissociation constant provides a quantitative measure of the ligand–receptor binding affinity, with lower Kd values indicating stronger interactions. The molecular docking results show that three ligands—oleanolic acid, alpha-peltatin, and camptothecin—exhibited more favorable docking energies compared to Neratinib, the established control. These compounds not only demonstrated more negative docking energies but also resulted in lower Kd values, suggesting that their binding to HER2 is more effective than that of Neratinib. This finding shows the potential therapeutic advantages of these ligands, particularly in targeting HER2 for cancer treatments. Their superior affinity indicates they could offer enhanced efficacy, possibly making them valuable candidates for further drug development in breast cancer therapy [54].

The results of the molecular docking analysis for the various ligands interacting with the EGFR are presented in Table 1. In comparison to the HER2 receptor, none of the ligands achieved favorable binding energies with EGFR, indicating a potential selectivity of these compounds toward HER2. This differential binding behavior suggests that these ligands may have a higher affinity for HER2, making them more effective in targeting that receptor. The results show a strong binding affinity between camptothecin and the HER2 receptor, as evidenced by favorable docking energy values compared to other compounds such as Neratinib, a clinically used HER2 inhibitor [55]. This finding suggests that camptothecin may exhibit higher selectivity for HER2-positive breast cancer, providing a targeted approach to treat this aggressive subtype.

Among the ligands, camptothecin stood out, exhibiting the most favorable binding energy with EGFR. This suggests that, despite the overall lower affinities compared to HER2, camptothecin retains a notable interaction with the EGFR. In contrast, alpha-peltatin did not perform as well, with binding energy values similar to the compound used as a reference, Neratinib. While most of the ligands showed binding energies close to that of Neratinib, only camptothecin demonstrated a greater affinity than the reference drug. This distinction makes camptothecin a particularly promising candidate for further investigation, as it demonstrated superior binding properties with both HER2 and EGFRs, potentially broadening its therapeutic applications.

#### 3.1.2. Molecular Interactions Between Camptothecin and HER2

**Figure 1:**
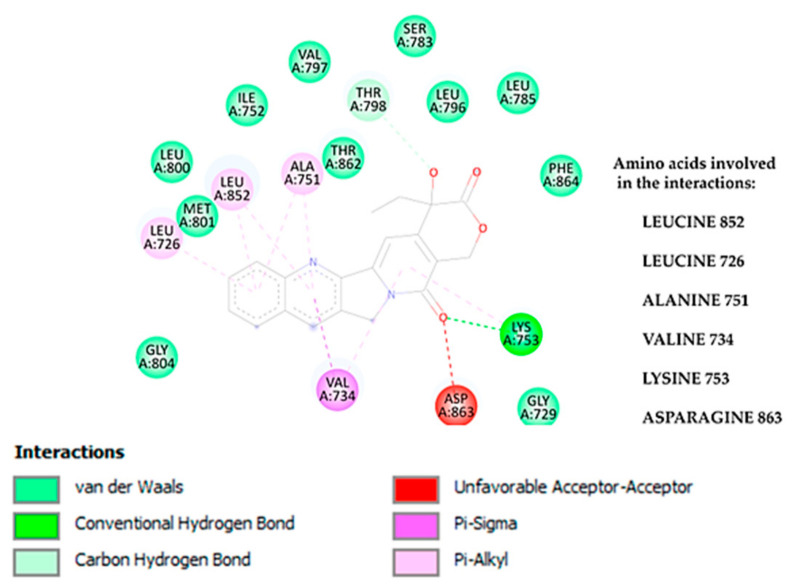
Two-dimensional interaction diagram between camptothecin and the HER2 receptor. Two-dimensional interaction diagram illustrating the interactions between camptothecin and the HER2 receptor reveals several key interactions. Hydrogen bonds are formed with the amino acid Lys753, while van der Waals interactions involve Met801, Thr862, Phe864, and Gly804. Additionally, pi–alkyl interactions are noted with Leu726, Leu852, and Ala751, and a pi–sigma interaction is observed with Val734.

Figure 1 illustrates the diverse interactions observed between camptothecin and the HER2 receptor in a detailed 2D interaction diagram. This figure identifies the specific amino acids that interact with camptothecin and classifies the types of molecular interactions generated. The predominant interactions are hydrophobic in nature, which is largely due to the structural characteristics of camptothecin, a molecule with several aromatic rings conducive to such non-polar interactions. Although camptothecin possesses functional groups capable of forming hydrogen bonds, only a few hydrogen bond interactions were observed in this case, specifically with the amino acid residues Lys 753 and Asp 863.

Additionally, certain amino acids such as Ala 751, Leu 852, Leu 726, and Val 734 engage in Pi–sigma and Pi–alkyl interactions with camptothecin, highlighting the contribution of aromatic and aliphatic amino acids to these non-covalent interactions. These interactions play a key role in stabilizing the ligand within the receptor binding site. The remaining amino acids, represented in green, are involved in van der Waals forces, which further enhance the overall binding stability. Although the van der Waals interactions are weaker than hydrogen bonds, Pi–sigma, and Pi–alkyl interactions, they are critical for ensuring a firm attachment of camptothecin within the HER2 receptor’s binding pocket. The combination of these interactions suggests a strong and stable affinity between camptothecin and the HER2 receptor, a factor that might contribute to the compound’s potential therapeutic efficacy.

**Figure 2:**
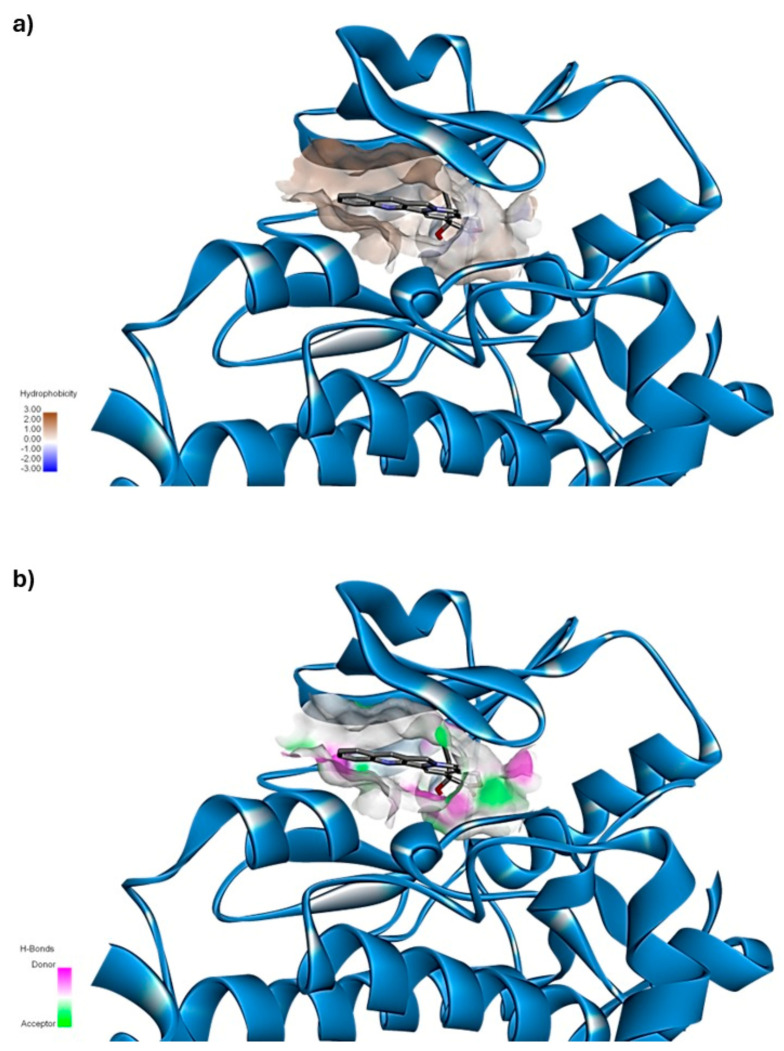
Interactions between camptothecin and HER2. The interactions identified include (**a**) hydrophobic interactions (represented by a brown surface), primarily with the amino acids Met801, Thr862, Phe864, and Gly804, along with a pi–sigma interaction observed with Val734 and (**b**) hydrogen bond interactions (depicted by a green proton-accepting surface and a pink proton-donating surface) primarily formed with the amino acid Lys753. Additionally, pi-alkyl interactions are noted with Leu726, Leu852, and Ala751.

To better illustrate the predominant types of interactions between the ligand and receptor, Figure 2 shows two complementary diagrams. Diagram (a) shows the protein surface, with areas shaded in brown to indicate hydrophobic regions. The intensity of the brown color correlates with the degree of hydrophobicity, where the darker shades represent amino acids that possess either cyclic or aliphatic side chains, contributing to a stronger hydrophobic environment. This hydrophobic nature suggests that the receptor surface predominantly consists of non-polar amino acids, which are more likely to engage in hydrophobic interactions with the ligand.

In contrast, diagram (b) highlights the areas of the protein surface capable of forming hydrogen bonds. These regions are divided based on their ability to act as hydrogen bond donors or acceptors, providing a detailed view of potential polar interactions. By comparing both diagrams, it is evident that while some hydrogen bonding is possible, hydrophobic interactions dominate the binding interface between the ligand and receptor. The overlap of these diagrams confirms that the ligand engages primarily in non-polar, hydrophobic interactions, rather than polar or hydrogen-bonding ones, due to the chemical composition of the receptor surface. This visualization reinforces the conclusion that hydrophobic forces are the main driving force stabilizing the ligand–receptor complex, which is crucial for understanding the binding behavior and potential selectivity of the ligand.

**Figure 3:**
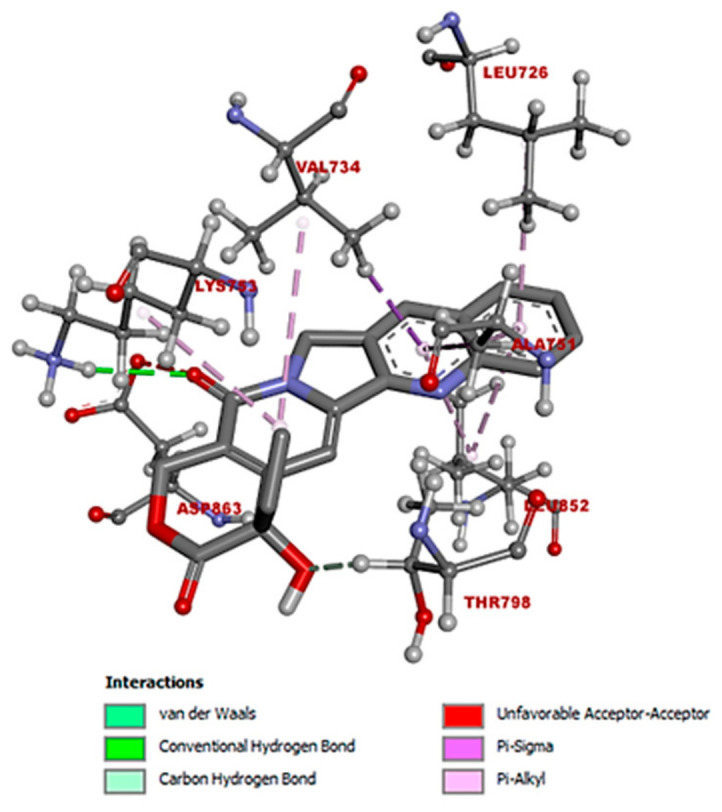
Amino acids involved in the interaction of camptothecin. The interaction with the different amino acids at a distance less than 4 Å. (Angstroms). The ball-and-stick graphic represents the amino acids of the protein, while the tube representation illustrates the ligand. The dotted lines indicate which atoms of the protein are interacting with the ligand. The color code is as follows: white represents hydrogen atoms, gray stands for carbon, purple denotes nitrogen, and red indicates oxygen. Hydrogen bonds are formed with the amino acid Lys753, while van der Waals interactions involved Met801, Thr862, Phe864, and Gly804. Additionally, pi-alkyl interactions are noted with Leu726, Leu852, and Ala751, and a pi–sigma interaction is observed with Val734.

Figure 3 provides a detailed visualization of the spatial arrangement of camptothecin and the specific amino acids with which it interacts, highlighting the nature of the bonds formed during the interaction. In this model design, camptothecin is depicted using a tube model to clearly illustrate the backbone structure of the molecule, facilitating an understanding of its conformation. The amino acids involved in the interaction are shown using a ball-and-stick model, where the different atoms within the amino acid side chains are represented with spheres and sticks, allowing for an easy discrepancy of each atom’s position and bonding relationship. In addition, the color-coding scheme used follows the standard conventions for atomic visualization: oxygen atoms are highlighted in red, nitrogen atoms in blue, hydrogen atoms in white, and carbon atoms in gray. This universally recognized color scheme enhances the readability of the structure, making it simple to identify the individual atoms and their role in forming various types of interactions, such as hydrogen bonds or van der Waals forces. Through this model design, the geometry and orientation of camptothecin relative to the receptor’s amino acids are effectively conveyed, providing a clear view of how the ligand fits within the receptor’s binding pocket. This figure also underscores the importance of the 3D conformation in determining the affinity and stability of the ligand–receptor complex, highlighting specific regions where bonds, such as hydrogen bonds or hydrophobic interactions, are likely to occur.

#### 3.1.3. Molecular Interactions Between Camptothecin and EGFR

**Figure 4:**
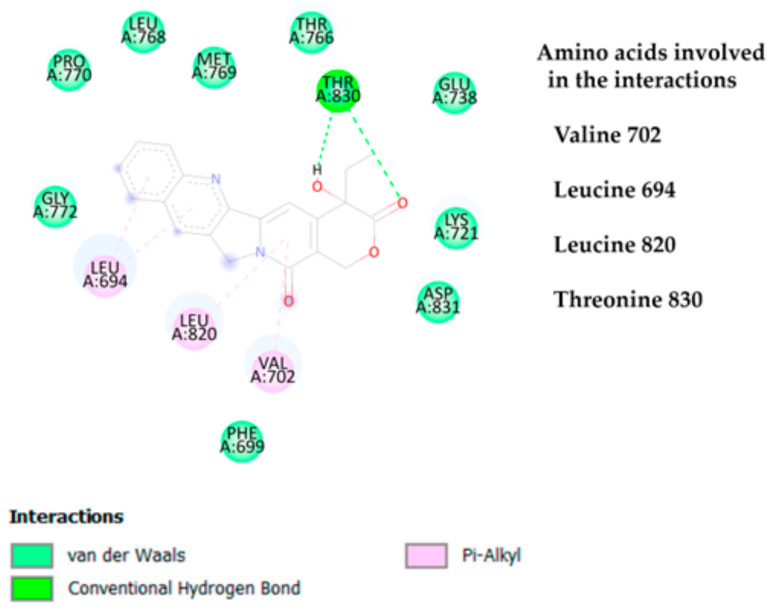
Two-dimensional interaction diagram between camptothecin and the EGFR. Hydrogen bond, van der Waals, pi-alkyl, and pi–sigma are the interactions obtained between camptothecin and the EGFR.

The results of camptothecin binding with the EGFR yielded less favorable binding energies in comparison to the interactions observed with the HER-2 receptor. As illustrated in Figure 4, camptothecin’s binding mode with the EGFR involves various interactions with key amino acid residues. Specifically, the residue Tyrosine 830 plays a critical role by forming hydrogen bond interactions with camptothecin, which likely contributes to the overall stabilization of the ligand–receptor complex, despite the relatively weaker binding affinity.

In addition to hydrogen bonding, hydrophobic interactions predominated in the interaction profile of camptothecin with the EGFR. Amino acid residues such as Leucine 694, Leucine 820, and Valine 702 primarily engage in pi–alkyl interactions with camptothecin. These pi–alkyl interactions may be due to the structural nature of camptothecin, which consists predominantly of aromatic rings. Aromatic rings typically promote hydrophobic interactions with the side chains of these amino acids, particularly in environments where pi–alkyl stacking is common. As a result, camptothecin establishes a network of primarily hydrophobic interactions with the receptor.

Furthermore, van der Waals forces are present but less dominant compared to hydrophobic and hydrogen bonding interactions, which align with the expectations based on the ligand’s structure. This set of interactions, however, could be insufficient to achieve a binding energy as favorable as that seen with the HER-2 receptor, suggesting that camptothecin’s selectivity and affinity are more pronounced towards HER-2. These findings reinforce the notion that the receptor’s binding pocket architecture and the specific amino acids involved significantly impact the binding strength and stability of the ligand–receptor complex.

**Figure 5:**
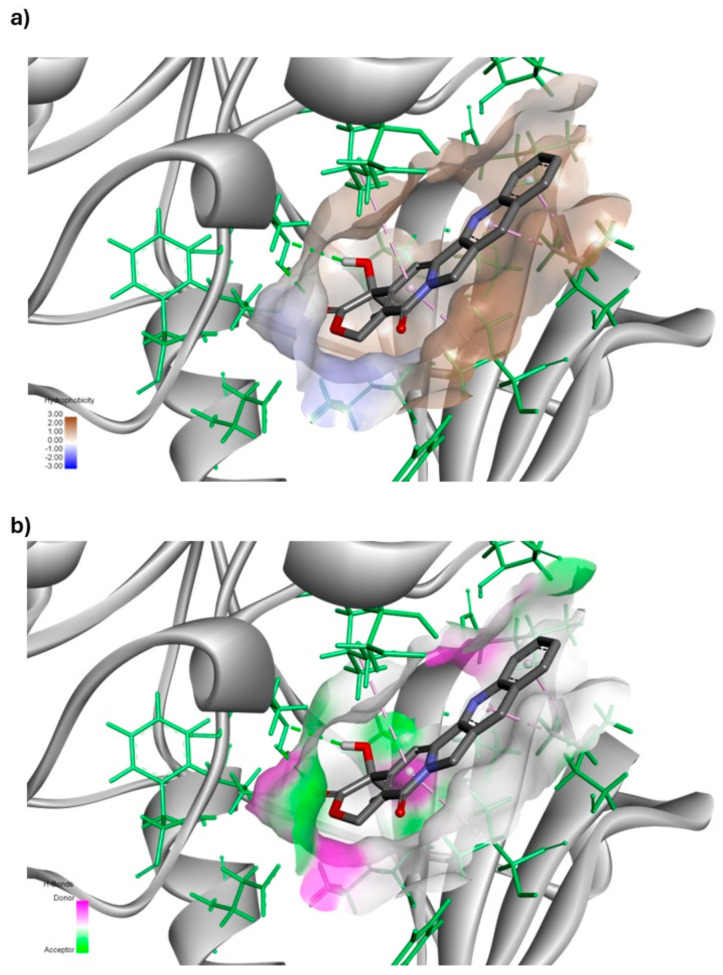
Interactions between camptothecin and the EGFR. The interactions obtained between camptothecin and the EGFR are (**a**) hydrophobic interactions (brown surface); (**b**) hydrogen bond interactions (green proton-accepting surface and pink proton-donating surface).

The areas of interaction, particularly in the right region and the left loop, where camptothecin engages in hydrophobic and electrostatic interactions, respectively, with the EGFR are shown in Figure 5. On the right, hydrophobic interactions predominate, which aligns with the structural properties of camptothecin, as it possesses several aromatic rings that readily engage in such interactions. These aromatic regions allow for strong pi–alkyl interactions with the hydrophobic residues of the receptor. The presence of such interactions is pivotal because they help to stabilize the ligand within the binding pocket through non-polar contacts, enhancing the overall ligand–receptor affinity.

On the left side of the diagram, the electrostatic interactions predominate, particularly hydrogen bonds. Although camptothecin is primarily hydrophobic, it contains polar functional groups that contribute to hydrogen bonding. These polar sections of the molecule are oriented towards the central binding region of the EGFR, where they form hydrogen bonds, most notably with Threonine 830. This hydrogen bonding interaction is crucial as it further stabilizes the camptothecin–receptor complex by anchoring the ligand in a precise position within the binding site. These combined hydrophobic and hydrogen bond interactions highlight the dual nature of camptothecin’s binding mechanism, with the aromatic regions primarily interacting through hydrophobic forces and the polar regions forming key electrostatic interactions.

This balance of interaction types underscores the complexity of ligand–receptor binding, where the distinct structural features of a molecule like camptothecin play a significant role in determining the strength and specificity of its binding with different regions of the receptor.

**Figure 6:**
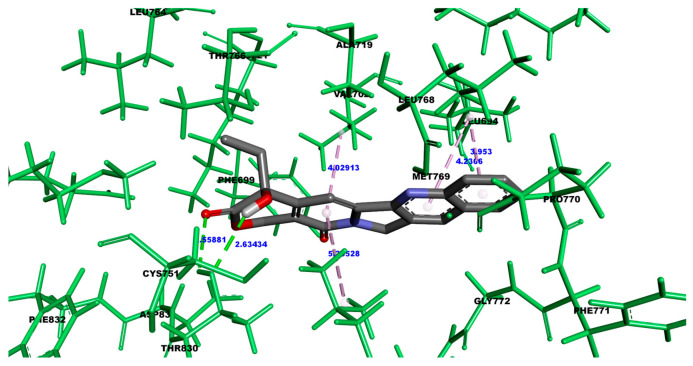
Distance of the interactions between camptothecin and the EGFR. The interaction with the different amino acids is at a distance less than 4 Å. A hydrogen bond typically requires a distance of less than 4 Å, as the electrostatic interaction between the hydrogen donor and acceptor significantly diminishes at greater distances.

The specific amino acids of the EGFR that interact with camptothecin and the bond distances measured in angstroms are shown in Figure 6. It is important to note that in molecular docking studies, hydrogen bonds are typically considered to occur at distances less than 4 Å, which indicates close proximity and potential for stronger interactions between the ligand and the receptor. The bond distances observed here provide insights into the spatial relationships that govern these interactions.

However, it must be emphasized that this particular docking study was carried out under the assumption that the protein, the EGFR, would remain rigid, while only the ligand, camptothecin, was modeled as flexible, allowing it rotational freedom. This is due to current limitations in considering the entire flexible system, such as the genetic algorithm in the Autodock Tools software, which allows a maximum of five flexible amino acids. In this study, we aimed to analyze the system without focusing on a specific site to identify the area of greatest affinity. Using a rigid system imposes the limitation that neither the ligand nor the amino acid residues of the protein can move. As a result, the findings are approximate compared to those that would be obtained from a fully flexible system. Therefore, it is crucial to complement these studies with molecular dynamics simulations. This approach simplifies the model but may not fully capture the dynamics of the protein–ligand interaction in a real biological environment. Both the ligand and the receptor are subject to constant movement and conformational changes due to thermal fluctuations and the dynamic nature of biological systems. Such flexibility in both the receptor and ligand is expected to lead to a greater number of interactions, which could significantly influence the overall binding affinity and stability of the complex.

Considering the dynamic environment of a biological system, where proteins and ligands are in continuous motion, it is plausible that more interactions would form over time, contributing to a more comprehensive understanding of the binding mechanism between camptothecin and EGFR. This would enhance our knowledge of how these interactions stabilize the ligand within the binding pocket and how they could play a critical role in the efficacy of camptothecin as a therapeutic agent.

#### 3.1.4. Molecular Dynamic (MD) Simulation

After performing the molecular docking, the conformation that exhibited the best docking energy was selected for further analysis and saved in PDB format to facilitate the subsequent molecular dynamics simulation. The simulation was conducted using GROMACS, with default parameters, a controlled temperature of 36 °C, and a simulation time of 100 nanoseconds. The parameters were chosen to mimic physiological conditions and allow an accurate evaluation of the ligand–receptor complex’s behavior over time.

**Figure 7:**
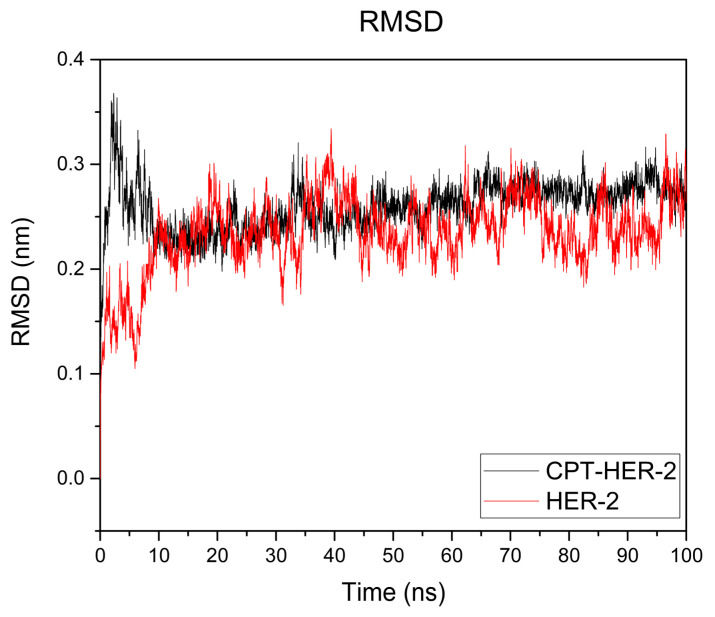
RMSD (root mean square deviation) graph at 100 nanoseconds between camptothecin and the HER2 receptor. The graph shows the stability of the protein–ligand complex (black line) and stability of HER-2 alone (red line).

Figure 7 illustrates the root mean square deviation (RMSD) graph obtained for the camptothecin–HER2 complex (black line) and the HER2 protein alone. The RMSD is a key indicator used to assess the structural stability of a protein–ligand complex during a molecular dynamics simulation. In this case, the graph shows that after approximately 10 nanoseconds (ns), the system reached a certain degree of equilibrium, suggesting that the complex achieved a relatively stable conformation. Examining both curves reveals that there are no significant fluctuations when comparing the protein complex to the protein alone, as no variation exceeds 0.5 nanometers. However, the curve representing the complex shows greater stability than that of HER2 alone.

**Figure 8:**
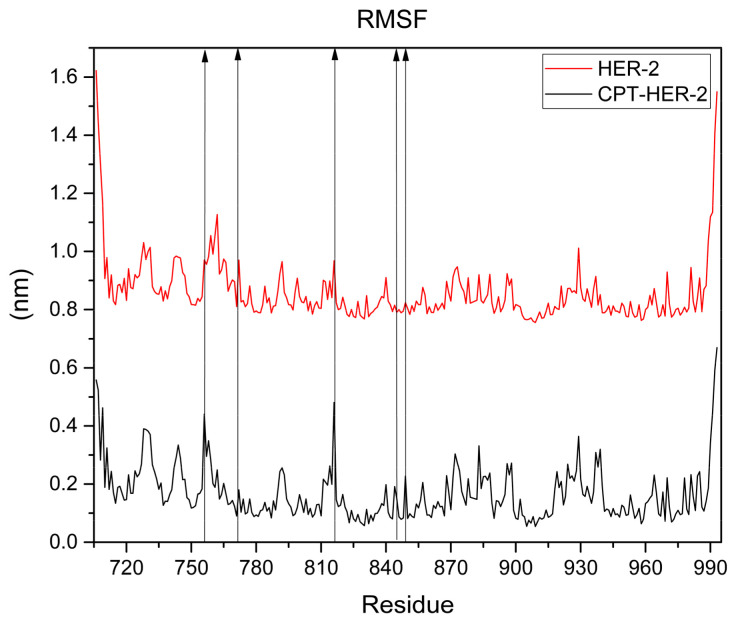
RMSF (root mean square fluctuation) graph at 100 nanoseconds between camptothecin and the HER2 receptor. The graph shows the stability of the protein–ligand complex (black line) and stability of HER-2 alone (red line).

Notably, throughout the 100 nanoseconds of the simulation, there were no significant fluctuations in the RMSD, which further supports the hypothesis that the binding interaction between camptothecin and the HER2 receptor remains stable over time. This consistency implies that the ligand is well fitted within the binding pocket of the HER2 protein and that the interactions formed are robust enough to maintain the structural integrity of the complex. Additionally, the simulation does not indicate any significant conformational changes in the HER2 protein during the evaluated period, suggesting that the binding of camptothecin does not induce major alterations in the protein’s structure. This is supported by Figure 8, which displays the RMSF graph for the HER2–camptothecin complex (black line) and HER2 alone (red line). The graph illustrates the fluctuations of each amino acid residue in the HER2 protein. Notably, only the amino acid residues bonded to camptothecin show negligible fluctuations, measuring less than 0.5 nm, indicating that the protein maintains structural stability. These findings reinforce the potential of camptothecin as a stable ligand for HER2 targeting in breast cancer therapy.

**Figure 9:**
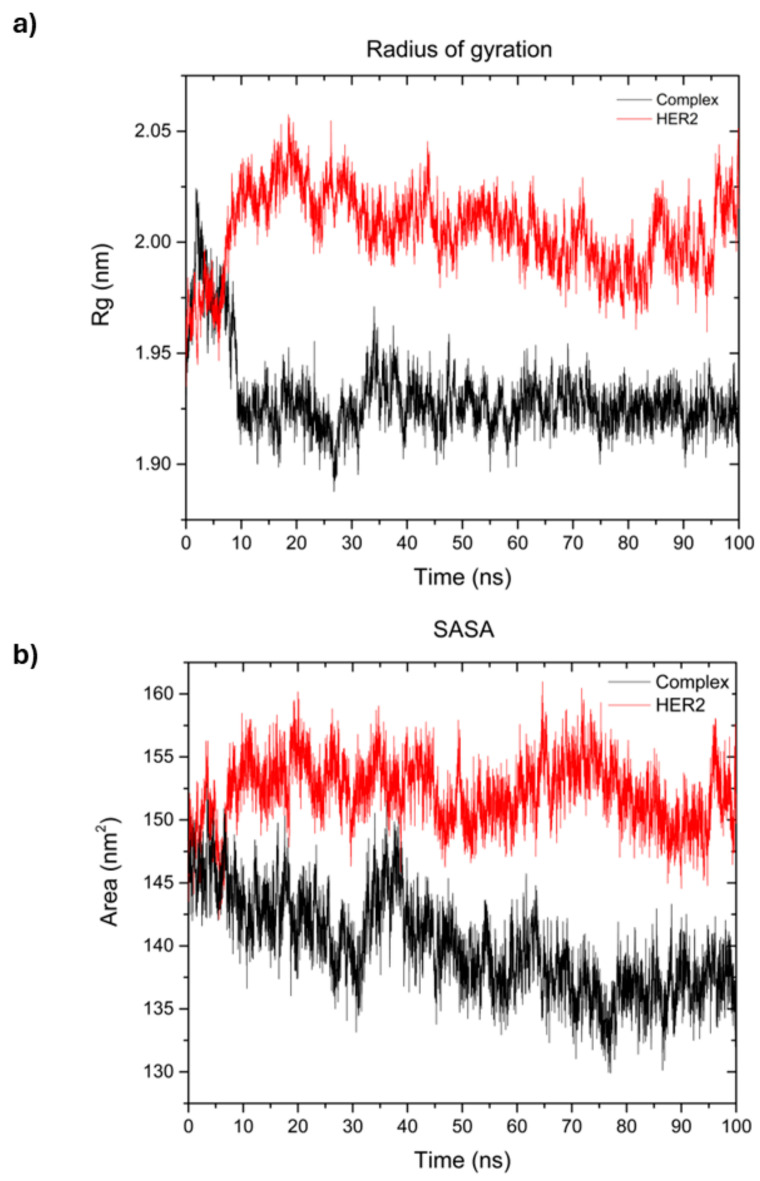
The stability of the protein and complex is assessed through the analysis of (**a**) the radius of gyration (Rg), where the red line represents the HER2 protein and the black line denotes the HER2–camptothecin complex, and (**b**) the solvent accessible surface area (SASA), with the red line indicating HER2 and the black line representing the HER2–camptothecin complex.

To determine if the binding of camptothecin affected the protein’s folding, we plotted the radius of gyration (Rg). This is illustrated in Figure 9, (a) where the red line represents HER2, and the black curve depicts the protein–ligand complex. The red curve representing HER2 alone shows that after the initial 10 nanoseconds, the protein’s compactness remains relatively stable, averaging a Rg of 2 nm. In contrast, the curve for the complex reveals that, similar to the HER2 receptor, the protein’s compactness remains consistent after the first 10 nanoseconds, averaging a Rg of 1.9 nm throughout the 100 ns simulation. This indicates that the protein remains more stable and folded, showing less variability than the HER2 protein alone. The lower average value of the complex, in comparison to the protein alone, suggests that there are no structural or folding changes occurring in the protein. This indicates that the complex remains stable throughout the duration of the simulation. Panel (b) displays the analysis of the solvent accessible surface area (SASA) to assess the protein’s stability. Higher values in nm^2^ suggest a loss of structural relaxation, indicating reduced stability in the protein. The red curve for the HER2 protein alone shows an average value of 155 nm^2^. The black curve represents the camptothecin–HER2 complex, which recorded an average value of 142 nm^2^. This indicates that the protein exhibits better stability when the complex is formed compared to the protein alone, even though the values of both the protein and the complex are relatively similar for both the Rg and SASA results. Molecular dynamics simulations demonstrated the stability of the camptothecin–HER2 complex, further supporting its potential as a therapeutic agent.

#### 3.1.5. Pharmacological Properties

Evaluating the pharmacological and physicochemical properties of compounds is essential to predict their behavior in the human body. ADME (Absorption, Distribution, Metabolism, and Excretion) characteristics provide insight into how a compound might be processed biologically, while physicochemical properties such as Log P (partition coefficient), molecular weight, and the number of hydrogen bond donors and acceptors are equally critical. These factors collectively determine the potential bioavailability, permeability, and overall efficacy of a compound as a drug.

Lipinski’s rule of five offers a useful framework for predicting drug-likeness, focusing on the aforementioned properties. This rule suggests that compounds are more likely to be orally active if they meet specific criteria, including, a Log P value below 5, molecular weight under 500 Daltons, fewer than five hydrogen bond donors, and fewer than ten hydrogen bond acceptors. These parameters are indicative of the ability of the compounds to permeate cell membranes and remain pharmacologically effective in the body.

The results of the evaluation of the ligands based on Lipinski’s rules, showing which ligands achieved the rule and which did not, are shown in Table 2. Notably, three ligands—camptothecin, acronycine, and alpha-peltatin, complied fully with Lipinski’s criteria, indicating a promising potential for bioavailability and interaction within the human body. Their compliance suggests that these compounds could behave favorably during drug absorption and distribution, providing a competitive advantage in therapeutic applications.

In contrast, neratinib, a commercially available drug used in breast cancer treatment, violated some of these rules, although it remains effective in clinical settings. This suggests that while Lipinski’s rules provide a strong foundational guide, there may be exceptions where pharmacokinetic optimization or other molecular modifications allow a drug to be effective despite not accomplishing all of these guidelines. Thus, the compounds that fully achieved the rule of five, camptothecin, acronycine, and alpha-peltatin, are promising; however, further studies and modifications may enhance their therapeutic potential. Table 3 presents a theoretical evaluation of several pharmacokinetic parameters, revealing similar results for all the evaluated ligands. It is clear that, due to their structural characteristics, they are fully soluble in water, which suggests potential challenges for efficient distribution within the body. However, their lipophilic traits allow for good absorption. Additionally, none of the ligands, except for acronycine, possess the ability to cross the blood–brain barrier, which is crucial for preventing side effects in the brain. Conversely, camptothecin was theoretically found not to inhibit any of the cytochromes, which is important for its proper metabolism and elimination from the body. It is important to note that these results are theoretical and require validation through experimental studies. Nonetheless, when compared to all the ligands, camptothecin exhibited the best results in terms of compliance with Lipinski’s rules and the pharmacokinetic parameters evaluated.

## 4. Discussion

In silico studies play a crucial role in the development of new medications for various diseases, despite certain limitations. For instance, these studies often fail to account for factors present in real-world systems, such as the influence of other substances, changes in volume, and fluctuating pressure. This is primarily because the calculations are based on rigid systems that utilize constant variables. Moreover, they do not consider the potential adverse effects that each substance may cause. Nevertheless, by comparing the proposed ligands to existing drugs under identical parameters, it is possible to derive approximations that help identify the most promising candidates for further in vitro and in vivo studies. In this context, molecular docking results indicate that camptothecin is the most promising candidate, demonstrating a higher affinity for both receptors than neratinib, a drug used for breast cancer treatment. Among all the evaluated compounds, camptothecin theoretically binds effectively to both proposed receptors, which are recognized to be present in the cell membrane. The observed interactions and binding distances suggest stability, attributed to the formation of hydrogen bonds with nearby polar amino acids and the molecule’s orientation towards hydrophobic amino acids with substituents. This creates an amphipathic molecule positioned in a stable manner within the protein’s environment. This study presents the results of molecular dynamics simulations conducted on HER2, which exhibited the highest affinity. The simulation, performed over 100 nanoseconds under standard pressure and temperature conditions, showed no significant fluctuations over time. After the initial 10 nanoseconds, the system stabilized, with fluctuations remaining below 0.5 nm. Additionally, a comparison of receptor dynamics alone (without any coupled ligands, aside from ions used to balance the overall charge) revealed similar curves for both systems. This suggests that the bound ligand does not induce structural changes in the protein. The RMSF (root mean square fluctuation) graph illustrates the fluctuations recorded over a 100-nanosecond simulation for both systems, showing that amino acids exhibited fluctuations of less than 0.5 nm. This suggests no significant structural changes in the protein. Notably, fluctuations were observed in the amino acids interacting with the ligand (LEU 852, LEU 726, ALA 751, VAL 734, LYS 753, ASP 863), which is expected since these residues are not bound to any ligand in the receptor alone, rendering them more stable. The radius of gyration analysis revealed that the complex had a lower compactness value compared to the protein alone. Similarly, the SASA (solvent accessible surface area) analysis showed that the complex exhibited a lower average value than the protein itself. These findings indicate that the protein’s folding remains unchanged, maintaining stability throughout the 100-nanosecond simulation period. Regarding Lipinski’s rule, camptothecin received a favorable assessment alongside the simulation of pharmacokinetic parameters. However, its low solubility poses a significant challenge, compounded by difficulties in obtaining it through synthetic methods or natural sources due to lengthy and costly purification processes. Low solubility has significant implications beyond cancer treatments, as it directly impacts the absorption and distribution of various drugs. Therefore, predicting potential solubility and other pharmacokinetic parameters assists us in identifying an effective administration route as well as the use of a vehicle or nanocarrier system to enhance these properties. An alternative approach could involve leveraging nanotechnology to enhance its therapeutic efficiency. Current studies are exploring camptothecin bound to nanoparticles for various diseases. However, using it as a targeting agent for HER2 or EGFRs presents a promising opportunity, given the limited information available on this compound in the context of breast cancer. The results indicate that camptothecin may serve as a potential anticancer agent; however, in silico findings must be validated through in vitro and in vivo studies to confirm its effects. While theoretical studies can predict possible therapeutic benefits, they do not account for real-world factors, such as the presence of other substances and receptors. Additionally, standard conditions are assumed in these studies, whereas actual environments can vary significantly. Further in vitro and in vivo studies are required to evaluate the effects of camptothecin directly on breast cancer cell lines and demonstrate its potential anticancer activity. Additionally, tests on normal cell lines should be conducted to assess toxicity and ensure safety for therapeutic applications.

## 5. Conclusions

This study underscores the significant potential of several ligands, particularly camptothecin, alpha-peltatin, and acronycine, in binding to HER2 with higher affinity than neratinib, a commercial drug used for breast cancer treatment. Molecular docking results revealed that camptothecin achieved the lowest binding energy (−8.3 kcal/mol) among all ligands evaluated, demonstrating superior affinity compared to neratinib (−7.1 kcal/mol). Additionally, pharmacokinetic analyses based on Lipinski’s rules confirmed that camptothecin, alpha-peltatin, and acronycine fully complied with all criteria, suggesting favorable ADME (Absorption, Distribution, Metabolism, and Excretion) properties and a high potential for bioavailability and permeability. The molecular dynamics simulations further validated the stability of the camptothecin–HER2 complex over 100 nanoseconds, with root mean square deviation (RMSD) fluctuations consistently remaining below 0.5 nm after the initial 10 nanoseconds. The radius of gyration (Rg) analysis indicated a more compact structure for the complex, with an average value of 1.9 nm, compared to 2.0 nm for HER2 alone. The solvent accessible surface area (SASA) analysis also revealed a lower average value for the complex (142 nm^2^) than the receptor alone (155 nm^2^), highlighting enhanced stability and reduced structural relaxation in the protein upon ligand binding. The selectivity exhibited by these ligands towards HER2, in contrast to the EGFR, suggests a promising approach for targeted therapy, minimizing potential off-target effects. Hydrophobic interactions and hydrogen bonding with key amino acids, including LEU 852, LEU 726, and ALA 751, contribute to the stability and efficacy of the camptothecin–HER2 complex. It is essential to carry out additional studies involving various breast cancer cell lines to assess how the findings from this research correlate with in vitro or in vivo studies. If the results are favorable, new approaches to address the challenges associated with camptothecin can be explored, such as alternative administration routes or the application of nanoparticles.

## Figures and Tables

**Table 1:** Results obtained from the coupling energies and the affinity constant between the ligands and HER2 and EGFRs.

Ligand	HER2		EGFR	
	Energy (kcal/mol)	Kd	Energy (kcal/mol)	Kd
Neratinib	−7.92	1.57 μM	−5.53	88.20 μM
Oleanolic Acid	−8.17	1.02 μM	−5.98	41.67 μM
Ursolic Acid	−7.91	1.60 μM	−5.81	54.76 μM
Acronine/Acronycine	−7.96	1.47 μM	−5.68	68.37 μM
α-Peltatin	−8.09	1.17 μM	−4.38	619.16 μM
Camptothecin	−8.03	1.30 μM	−6.00	40.01 μM
Panaxadiol	−7.99	1.39 μM	−5.18	158.94 μM

**Table 2:** Evaluation results based on Lipinski’s rules indicate that the analyzed compounds must satisfy criteria related to molecular weight, the number of hydrogen bond donors and acceptors, and log P, which suggest a favorable potential for oral absorption and bioavailability. Additionally, the table outlines how many criteria are not met for each compound (No. violations), helping to identify the candidate with the most optimal parameters.

Ligand	Log p	MW	nOHNH	nON	No. Violations
Camptothecin	2.03	348.36	1	6	0
Neratinib	5.33	557.05	2	9	2
Oleanolic Acid	6.72	456.71	2	3	1
Ursolic Acid	6.79	456.71	2	3	1
Acronycine	3.85	321.38	0	4	0
α-Peltatin	1.74	400.38	2	8	0
Panaxadiol	6.98	460.74	2	3	1

MW: Molecular weight; nOHNH: proton donor; nON: proton acceptor; No. violations: number of violations.

**Table 3:** Results derived from the prediction of various pharmacokinetic parameters.

Ligand	Water Solubility	GI Absorption	Lipophilicity	CYP Inhibitor	BBB Permeant
Camptothecin	Moderately soluble	High	2.20	No	No
Neratinib	Poorly soluble	Low	4.24	Yes	No
Oleanolic Acid	Poorly soluble	Low	6.07	No	No
Ursolic Acid	Poorly soluble	Low	5.93	No	No
Acronycine	Moderately soluble	High	3.43	Yes	Yes
α-Peltatin	Moderately soluble	High	2.72	Yes	No
Panaxadiol	Poorly soluble	High	5.90	No	No

GI: gastrointestinal; BBB: blood–brain barrier.

## Data Availability

All data reported is available under request.
